# Phenotypic and Genomic Analysis of Hypervirulent Human-associated *Bordetella bronchiseptica*

**DOI:** 10.1186/1471-2180-12-167

**Published:** 2012-08-06

**Authors:** Umesh Ahuja, Minghsun Liu, Shuta Tomida, Jihye Park, Puneet Souda, Julian Whitelegge, Huiying Li, Eric T Harvill, Julian Parkhill, Jeff F Miller

**Affiliations:** 1 Department of Microbiology, Immunology and Molecular Genetics, University of California, BSRB 254, 615 Charles E. Young Drive East, Los Angeles, CA, 90095-1747, USA; 2 Department of Molecular and Medical Pharmacology, Crump Institute for Molecular Imaging, University of California, Los Angeles, USA; 3 The Pasarow Mass Spectrometry Laboratory, The NPI-Semel Institute, David Geffen School of Medicine, University of California, Los Angeles, USA; 4 Department of Veterinary and Biomedical Sciences, The Pennsylvania State University, Pennsylvania, USA; 5 Pathogen Sequencing Unit, Wellcome Trust Sanger Institute, Hinxton, Cambridge, UK

**Keywords:** *B. bronchiseptica*, Hypervirulence, Cytotoxicity, *Bordetella* evolution, Host adaptation, Pathogenomics

## Abstract

**Background:**

*B. bronchiseptica* infections are usually associated with wild or domesticated animals, but infrequently with humans. A recent phylogenetic analysis distinguished two distinct *B. bronchiseptica* subpopulations, designated complexes I and IV. Complex IV isolates appear to have a bias for infecting humans; however, little is known regarding their epidemiology, virulence properties, or comparative genomics.

**Results:**

Here we report a characterization of the virulence of human-associated complex IV *B. bronchiseptica* strains. In *in vitro* cytotoxicity assays, complex IV strains showed increased cytotoxicity in comparison to a panel of complex I strains. Some complex IV isolates were remarkably cytotoxic, resulting in LDH release levels in A549 cells that were 10- to 20-fold greater than complex I strains. *In vivo*, a subset of complex IV strains was found to be hypervirulent, with an increased ability to cause lethal pulmonary infections in mice. Hypercytotoxicity *in vitro* and hypervirulence *in vivo* were both dependent on the activity of the *bsc* T3SS and the BteA effector. To clarify differences between lineages, representative complex IV isolates were sequenced and their genomes were compared to complex I isolates. Although our analysis showed there were no genomic sequences that can be considered unique to complex IV strains, there were several loci that were predominantly found in complex IV isolates.

**Conclusion:**

Our observations reveal a T3SS-dependent hypervirulence phenotype in human-associated complex IV isolates, highlighting the need for further studies on the epidemiology and evolutionary dynamics of this *B. bronchiseptica* lineage.

## Background

Human pathogens often evolve from animal reservoirs, and changes in virulence sometimes accompany acquisition of the ability to infect humans [1]. Examples include smallpox virus, HIV, enterohemorrhagic *E. coli*, and *Bordetella pertussis*. Understanding how these events occur requires the ability to reconstruct evolutionary history, and this can be facilitated by the identification of evolutionary intermediates. An experimentally tractable opportunity to study human adaptation is provided by *Bordetella* species. The *Bordetella* genus currently includes nine closely related species, several of which colonize respiratory epithelial surfaces in mammals. *B. pertussis,* the etiological agent of pertussis (whooping cough) is exclusively adapted to humans; *B. parapertussis* refers to two groups, one infects only humans and the other infects sheep [2,3]; and *B. bronchiseptica* establishes both asymptomatic and symptomatic infections in a broad range of mammalian hosts, which sometimes include humans [4-7]. Numerous studies have implicated *B. bronchiseptica* as the closest common ancestor of human-adapted bordetellae, with *B. pertussis* and *B. parapertussis*_*hu*_, evolving independently from different *B. bronchiseptica* lineages [8-10]. The genomes of these 3 species differ considerably in size and *B. pertussis* and *B. parapertussis* have undergone genome decay, presumably as a consequence of niche restriction [6].

Most mammalian bordetellae express a common set of virulence factors which include putative adhesins such as filamentous hemagglutinin (FHA), fimbriae, and pertactin, and toxins such as a bifunctional adenylate cyclase/hemolysin, dermonecrotic toxin, and tracheal cytotoxin. *B. pertussis* additionally produces pertussis toxin [7]. Of particular significance here is the *bsc* type III secretion system (T3SS) locus which encodes components of the secretion machinery, associated chaperones, and regulatory factors. Remarkably, only a single T3SS effector, BteA, has been identified to date [11-13]. BteA is an unusually potent cytotoxin capable of inducing rapid, nonapoptotic death in a diverse array of cell types [14-16]. T3SS and *bteA*loci are highly conserved in *B. pertussisB. parapertussis*, and *B. bronchiseptica*[14,15].

A seminal phylogenetic analysis using multilocus sequence typing (MLST) of 132 *Bordetella* stains with diverse host associations led to the description of a new *B. bronchiseptica* lineage, designated complex IV, which differs in several respects from the canonical complex I *B. bronchiseptica* cluster [10]. Complex I strains are most commonly isolated from non-human mammalian hosts, whereas the majority of complex IV strains were from humans, many with pertussis-like symptoms. Complex IV strains were found to exclusively share IS1663 with *B. pertussis*, suggesting a close evolutionary relationship among these lineages. Complex IV strains and *B. pertussis* are proposed to share a common ancestor, although the genes encoding pertussis toxin (*ptxA-E*) and the *ptl* transport locus were found to be missing in the majority of complex IV strains that were sampled [10]. Additionally, several other *B. pertussis* virulence genes were also found to be absent or highly divergent, including those encoding dermonecrotic toxin, tracheal colonization factor, pertactin, and the lipopolysaccharide biosynthesis locus. Differences between virulence determinants expressed by *B. pertussis* and complex IV strains have been suggested to be driven by immune competition in human hosts [10], a model also proposed for differences observed between *B. pertussis* and *B. parapertussis*_*hu*_[17].

Given their apparent predilection of complex IV *B. bronchiseptica* isolates for human infectivity, we have initiated a systematic analysis of their virulence properties and mechanisms. We found that complex IV strains, on average, display significantly elevated levels of cytotoxicity in comparison to complex I isolates. Several complex IV strains are also hyperlethal in mice, and hyperlethality *in vivo* as well as cytotoxicity *in vitro* is dependent on the BteA T3SS effector protein [11,12]. Comparative whole-genome sequence analysis of four complex IV isolates was used to identify similarities and differences between *B. bronchiseptica* lineages. Results from genome comparisons did not identify significant genomic regions that are unique to complex IV strains but missing from complex I isolates. This implies that complex IV-specific phenotypes are determined by polymorphisms in conserved genes, differential regulation [18], or other epigenetic mechanisms rather than acquisition or retention of unique genomic determinants.

## Methods

### Bacterial strains and growth conditions

Strains and plasmids used in this study are listed in Table 1. Bacteria were grown in Stainer-Scholte liquid (SS) medium at 37°C [19] or on Bordet–Gengou (BG) agar (Becton Dickinson Microbiology systems) supplemented with defibrinated sheep blood at a concentration of 7.5% and incubated at 37°C. RB50 [20] was grown from archived, low passage, frozen glycerol stock. Antibiotics were added to the following final concentrations: ampicillin (Ap), 100 μg/ml; chloramphenicol (Cm), 25 μg/ml; Streptomycin (Sm), 20 μg/ml; Kanamycin (km), 50 μg/ml; Gentamycin (Gm), 20 μg/ml.

**Table 1:** Bacterial strains, mammalian cells and plasmids used in this study

**Bacterial strains or plasmids**	**Alternate name**	**Source**	**Genotype or relevant characteristics**	**Reference**
*E.coli* strains				
DH5α			SupE44λlacU169 (Φ80lacZλM15) hsdR17recA1 endA1 gyrA96 thi-1 relA1	[48]
SM10λpir			Expression of the π protein for replication of suicide vector	[49]
*Bordetella* strains				
RB50		Rabbit	Complex-I strain, ST-12,Sm^r^	[20]
RB50λ*bscN*	WD3		RB50λ*bscN*, Sm^r^	[15]
RB50λbteA			RB50λ*bteA*, Sm^r^	[11]
A309	SBL-F6116	Human	Complex-IV strain, ST-9, Sm^r^	[10]
A310	SBL-F6368	Human	Complex-IV strain, ST-8, Sm^r^	[10]
A345	GA96-01	Human	Complex-IV strain, ST-21, Sm^r^	[10]
Bbr69	591	Dog	Complex-IV strain, ST-22, Sm^r^	[10]
Bbr77	675	Human	Complex-IV strain, ST-18, Sm^r^	[10]
D444	MO149	Human	Complex-IV strain, ST-15, Sm^r^	[10]
D445	MO211	Human	Human complex-IV strain, ST-17, Sm^r^	[10]
D446	MO275	Human	Complex-IV strain, ST-3, Sm^r^	[10]
D758	00-P-2730	Human	Complex-IV strain, ST-34, Sm^r^	[10]
D445λ*bscN*			Complex-IV strain, ST-17, Sm^r^	This study
D445λ*bteA*			Complex-IV strain, ST-17, Sm^r^	This study
Bbr77λ*bscN*			Complex-IV strain, ST-18, Sm^r^	This study
Bbr77λ*bteA*			Complex-IV strain, ST-18, Sm^r^	This study
Bbr68	590	Dog	Complex-I strain, ST-10, Sm^r^	[10]
Bbr78	680	Koala bear	Complex-I strain, ST-7, Sm^r^	[10]
Bbr79	401	Dog	Complex-I strain, ST-7, Sm^r^	[10]
545		Pig	Complex-I strain, ST-7, Sm^r^	[10]
548		Pig	Complex-I strain, ST-4, Sm^r^	[10]
599		Dog	Complex-I strain, ST-27, Sm^r^	[10]
601		Dog	Complex-I strain, ST-4, Sm^r^	[10]
705		Rabbit	Complex-I strain, ST-10, Sm^r^	[10]
723		Cat	Complex-I strain, ST-23, Sm^r^	[10]
782		Cat	Complex-I strain, ST-5, Sm^r^	[10]
BBE001		Human	Complex I, ST-11	[34]
BBF579		Human	Complex IV, Novel ST	[34]
Mammalian cells				
HeLa/CCL-2^TM^			Human cervical adenocarcinoma cell line	ATCC
A549/CCL-185^TM^			Human lung carcinoma cell line	ATCC
J774A.1/ TIB-67^TM^			Mouse monocyte-macrophage cell line	ATCC
Plasmids				
pEGBR1005			pSS1129 based suicide plasmid harboring *bscN* in-frame deletion of codons 171–261, *pir* dependent, *oriT*, *oriV*, *sacB*, Km^R^	[15]
pRE112-λ*bteA*			pGP704 based suicide plasmid harboring *bteA* in-frame deletion of codons 4–653, *pir* dependent, *oriT*, *oriV*, *sacB*, Cm^R^	[11]
pBBR1MCS-5			*lacPOZ*’ *mob*^+^, broad-host- cloning vector, Gm^R^	[50]
p*bteA*			*bteA* cloned into pBBR1MCS-5, Gm^R^	[11]

### Sample preparation, protein electrophoresis and immunoblotting

For SDS-polyacrylamide gel electrophoresis (SDS-PAGE) sample preparation, bacteria were cultured in SS media overnight and harvested by centrifugation at 10,000 x *g* at 4°C for 10 min. The resulting supernatant, containing secreted proteins, was filtered through a 0.2-μm membrane to remove contaminating bacterial cells. Protein from supernatants (equivalent of 3.75 O.D._600_) was precipitated with 15% trichloroacetic acid (TCA) for 1 h on ice and samples were centrifuged at 15,000 *g* for 15 min at 4°C. After centrifugation, TCA was removed and the pellet was resuspended in 1 X SDS-loading dye with 25 mM freshly prepared DTT. To neutralize the acidic pH of the samples, a few crystals of tris-base were added. Protein pellets were dissolved by shaking over a bench-top shaker for 30 min at room temperature prior to fractionation by various fixed percentage or gradient (as indicated) pre-cast SDS-polyacrylamide gels (Bio-Rad). The pellet samples after normalization to 12.5 O.D._600_/ml, were boiled for 10 min in 1 x SDS-loading dye as above. After the run, proteins were either Coomassie stained or transferred onto a polyvinylidene difluoride (PVDF) membrane (Immobilon P, Millipore) using a semi-dry blot. BvgS, a non-secreted protein control was detected using polyclonal mouse antiserum at a dilution of 1:1000 [21]. Pertactin (PRN), which is secreted by a non-T3SS dependent pathway, was identified using a monoclonal mouse antibody at a dilution of 1:1000 [22]. Bsp22, a T3SS substrate control, was detected using polyclonal mouse serum at a dilution of 1:10,000 [23]. Immunodetection was carried out by chemifluorescence using horseradish peroxidase-labeled goat anti-mouse IgG and the ECL plus® detection substrate (GE Healthcare). Chemifluorescent signals were visualized using a Typhoon scanner (GE Healthcare).

### Genomic DNA extraction, PCR-based detection and genome sequencing

DNA was extracted from overnight cultures of various isolates using the PureLink genomic DNA kit as per manufacturer’s instructions (Invitrogen Corporation, USA). PCR was performed according to the manufacturer's instructions (0.5 U of iproof polymerase, 200 μM each of the four dNTPs and 1 μM each primer) and supplemented with 3% dimethyl sulphoxide (DMSO). Primers B77_QseC1F (5^′^- ATGACTTTGCAGCGCAGGTT −3^′^) and B77_QseC1R (5^′^- AGAAACGCGATCAGCACGGG −3^′^) or primers B77_QseC2F (5^′^- GGAGATCTTGCCGTCGCCAT-3^′^) and B77_QseC2R (5^′^-ACTTCCCATTGCGCGCGTAG-3^′^) were used to amplify *qseC* sequences, and primers B77_QseB1F (5^′^- GAGAATTCTTATTGTCGAAG-3^′^) and B77_QseB1R (5^′^- GATTCCCAGTCATACAGCTT −3^′^) were used to amplify *qseB*. Cycling parameters were: one cycle of 98°C for 1 min; 25 cycles of 98°C for 10 s, 55°C for 20 s and 72°C for 30 s; and a final incubation at 72°C for 5 min. The PCR products were fractionated on 1% agarose gel using 1X TBE buffer containing 5 μg/ml ethidium bromide. PCR products of the extracted DNA were then purified for sequencing using Qiagen's QIAquick purification kit (Qiagen, Valencia, USA). *Bordetella* genomes were sequenced by the Sequencing Group at the Sanger Center and can be obtained from ftp://ftp.sanger.ac.uk/pub/pathogens/bp.

### Construction of *bscN* and *bteA* in-frame deletion mutants

To construct in-frame deletions of codons 171–261 in the *bscN* locus, allelic exchange was performed using pEGBR1005 suicide plasmid derivatives as previously described by Yuk *et al. *[15]. For construction of *bteA* in-frame deletions (codons 4–653), suicide plasmid pRE112-*bteA* was used as previously described by Panina *et al. *[11]. All mutants were verified by sequencing target open reading frames.

### Cell lines

Cell lines used in this study were obtained from the American Tissue Culture Collection (ATCC). Human Cervical Adenocarcinoma, HeLa (ATCC CCL-2^TM^) and Mouse Monocyte-macrophage, J774A.1 (ATCC TIB-67^TM^) cell lines were maintained in Dulbecco's Modified Eagle Medium(DMEM), and Human Lung Carcinoma, A-549 cells (ATCC CCL-185^TM^) were maintained in Ham’s F-12 K medium (F-12 K) supplemented with 10% Fetal bovine serum (FBS) at 37°C with 5% CO_2._

### Cytotoxicity assays

Bacteria were cultured in SS media overnight and were then sub-cultured in SS media to an optical density of ~0.5 at 600 nm. For cytotoxicity assays, bacteria were added to previously seeded cell monolayers in 12- or 24-well tissue culture plates at the indicated MOIs. The plates were centrifuged for 5 min at 60 x g and incubated for up to 4 h at 37°C with 5% CO_2._ To measure cell cytotoxicity, Lactate dehydrogenase (LDH) release was used as a surrogate marker for cell death. LDH release in the supernatant media was assayed using a CytoTox 96® non-radioactive cytotoxicity assay kit (Promega, Madison, WI), according to the manufacturer's instructions. The maximal LDH release was defined as 100% and was determined by adding lysis solution to uninfected monolayers, determining the absorbance at 490 nm, and then subtracting the background value. Each sample was measured in triplicate in at least three independent experiments.

### Animal infection experiments

Wild-type female C57BL/6NCr (B6) mice, 4–6 weeks of age, were purchased from Charles River Breeding Laboratories (Wilmington, MA). The animals were lightly sedated with isoflurane (Novation Laboratories, TX) prior to intranasal infection with the indicated number of CFU of bacteria in a total volume of 40 μl of phosphate-buffered saline (PBS, Mediatech Inc, VA). Bacteria were cultured in SS media overnight and were then sub-cultured in SS media to an optical density of ~0.5 at 600 nm. Inocula were confirmed by plating serial dilutions. For survival curves, groups of four mice were inoculated with the indicated dose, and the percent survival was monitored over a 30-day period. Mice with lethal bordetellosis, indicated by ruffled fur, labored breathing, and diminished responsiveness, were euthanized to alleviate unnecessary suffering [24]. To enumerate the number of bacteria in respiratory organs, groups of three to four mice were sacrificed at the indicated time points and bacterial numbers in the lungs and tracheas were quantified by plating dilutions of tissue homogenates on BG plates with appropriate antibiotics, following incubation at 37°C for 2 days. The mean ± the standard error was determined for each group. The statistical significance between different groups was calculated by Student's two-tailed *t*-test. A significance level was set at *P* values of ≤0.05. All animal experiments were repeated at least three times with similar results. Murine survival percentage was analyzed with the Log-Rank (Mantel-Cox) test. All mice were maintained in UCLA animal research facilities according to National Institutes of Health and University of California Institutional Animal Care Committee guidelines. Animals were housed under specific pathogen-free conditions with free access to food and water. All experiments were approved by the UCLA Chancellor's Animal Research Committee.

### Histopathological **analysis**

Lungs were inflated with 10% neutral buffered formalin at the time of necropsy. Following fixation, tissue samples were embedded in paraffin, sectioned at 5 μm, and stained with hematoxylin-eosin, Giemsa, and Warthin-Starry for light microscopic examination at the Translational Pathology Core Laboratory of UCLA. Sections were scored for pathology by a veterinarian with training and experience in rodent pathology who was blinded to experimental treatment. The degree of inflammation was assigned an arbitrary score of 0 (normal = no inflammation), 1 (minimal = perivascular, peribronchial, or patchy interstitial inflammation involving less than 10% of lung volume), 2 (mild = perivascular, peribronchial, or patchy interstitial inflammation involving 10-20% of lung volume), 3 (moderate = perivascular, peribronchial, patchy interstitial, or diffuse inflammation involving 20-50% of lung volume), and 4 (severe = diffuse inflammation involving more than 50% of lung volume).

### *In vitro* adherence assays

Human lung epithelial (A549) cells and Human cervical epithelial (HeLa) cells were grown in F-12 K and DMEM medium, containing 10% fetal calf serum on cover slips in standard 12-well tissue culture plates, respectively. Bacteria in their mid-log phase were added to cell monolayers at a MOI of 200 as previously described [25]. The plates were spun at 200 × *g* for 5 min and then incubated for 15 min at 37°C. The cells were then washed six times with Hanks' balanced salts solution, fixed with methanol, stained with Giemsa stain (Polyscience, Warrington, PA) and visualized by light microscopy. Adherence was quantified by counting the total number of bacteria per eukaryotic cell in at least three microscopic fields from two separate experiments.

### Trypsin digestion of polypeptides for mass spectrometry

For secretome analysis by mass spectrometry, bacteria were cultured in SS media overnight and were then sub-cultured in SS media to an optical density at 600 nm of ~1.0. A 5 ml aliquot was removed and centrifuged at 10,000 x *g* at 4°C for 10 min to remove bacterial cells. The resulting supernatant, containing proteins secreted into the culture medium, was filtered through a 0.2 μm membrane to remove contaminating bacterial cells. The filtered supernatants were then desalted and concentrated using a centrifugal filter device (Amicon Ultra-3 K, Millipore) into ~300 μl of 50 mM ammonium bicarbonate buffer. The samples were reduced by incubation in 10 mM dithiotreitol (DTT) in 50 mM ammonium bicarbonate at 37°C for 1 h. They were then alkylated by adding 55 mM iodoacetamide in 50 mM ammonium bicarbonate and incubated at 37°C in dark for 1 h. Finally, the samples were digested at 37°C overnight with addition of 75 ng trypsin (EC 3.4.21.4, Promega) in 50 mM ammonium bicarbonate. For in-gel trypsin digestion of polypeptides, a previously described method was used [26].

### Nano-liquid chromatography with tandem mass spectrometry (nLC-MSMS)

nLC-MS/MS with Collision Induced Dissociation (CID) was performed on a linear trap quadrupole fourier transform (LTQ FT, Thermo Fisher, Waltham, MA) integrated with an Eksigent nano-LC. A prepacked reverse-phase column (Microtech Scientific C18 with a dimension of 100 μm x 3.5 cm) containing resin (Biobasic C18, 5-μm particle size, 300-Å pore size, Microtech Scientific, Fontana, CA) was used for peptide chromatography and subsequent CID analyses. ESI conditions using the nano-spray source (Thermo Fisher) for the LTQ-FT were set as follows: capillary temperature of 220°C, tube lens 110 V, and a spray voltage of 2.5 kV. The flow rate for reverse-phase chromatography was 5 μl/min for loading and 300 nl/min for the analytical separation (buffer A: 0.1% formic acid, 1% acetonitrile; buffer B: 0.1% formic acid, 100% acetonitrile). Peptides were resolved by the following gradient: 2–60% buffer B over 40 min, then increased to 80% buffer B over 10 min and then returned to 0% buffer B for equilibration of 10 min. The LTQ FT was operated in data-dependent mode with a full precursor scan at high-resolution (100000 at m/z 400) and six MSMS experiments at low resolution on the linear trap while the full scan was completed. For CID the intensity threshold was set to 5000, where mass range was 350–2000. Spectra were searched using Mascot software (Matrix Science, UK) in which results with p < 0.05 (95% confidence interval) were considered significant and indicating identity. The data was also analyzed through Sequest database search algorithm implemented in Discoverer software (Thermo Fisher, Waltham, MA).

### Identification of the core, non-core, and pan-genome of *Bordetella*

"Core" regions were defined as genome sequences that were present in all 11 *Bordetella* genomes, while "non-core" regions were defined as genome sequences that are not present in all genomes. RB50 was used as the reference genome. For each of the other 10 sequences, genomes were mapped to the reference genome using Nucmer [27]. All 10 “.coords” output files from the Nucmer program were analyzed to identify overlap regions based on RB50 coordinates using a Perl script. Finally, “core” sequences were extracted based on the genome sequence of RB50 with the coordinates calculated above. Unshared regions were then added to the reference genome to make a “revised” reference genome, which contained the original sequence plus unshared sequences. This process was repeated until all of the genomes were compared to include all unshared sequences included in the pan-genome. The core region was subtracted from the pan-genome of all the 11 genomes, and the remaining regions were identified as non-core regions.

### Hierarchical clustering using Cluster and Java Tree View

844 non-core fragments with more than 1000 bp were identified. An 844 row x 11 column matrix, in which 1 means "present" while 0 means "absent" for each non-core region, was entered to the Cluster program (http://bonsai.hgc.jp/~mdehoon/software/cluster/software.htm#ctv) [28]. Average linkage was used for clustering. The Java Tree View program [28] was used to show the clustering result.

## Results

### Hypercytotoxicity of complex IV isolates *in vitro*

**Figure 1:**
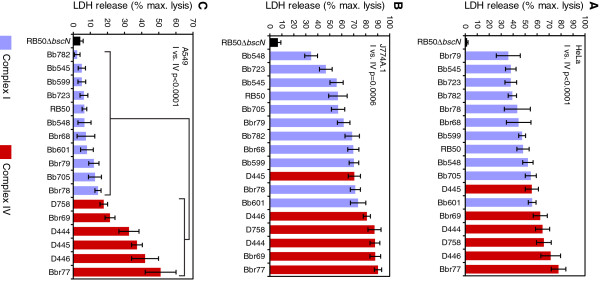
** Cytotoxicity of complex I and complex IV*B. bronchiseptica*isolates. A**. HeLa, **B**. J774A.1, or **C**. A549 cells were infected with the indicated strains at a multiplicity of infection (MOI) of 50 in 24-well plates for 3 h. Following infection, release of lactate dehydrogenase (LDH) into culture medium was measured as described in Materials and Methods. Complex I and complex IV strains are designated by blue or red bars, respectively. *P* values were calculated by an unpaired two-tailed Student's *t* test.

Cytotoxicity against a broad range of cell types is a hallmark of *B. bronchiseptica* infection *in vitro*[11,12,14,16,23]. To measure relative levels of cytotoxicity, human epithelial cells (HeLa), murine monocyte-macrophage derived cells (J774A.1), or human pneumocyte-derived cells (A549) were infected with an array of complex I or complex IV *B. bronchiseptica* isolates (Figure 1A-C). These strains represent different multilocus sequence types (STs), and they were isolated from both human and non-human hosts (Table 1). Lactate dehydrogenase (LDH) release was used as a surrogate marker for cell death, and RB50, an extensively characterized complex I rabbit isolate classified as ST12, was used as a positive control for cytotoxicity [20]. An isogenic RB50 derivative with a deletion in *bscN,* which encodes the ATPase required for T3SS activity [15], served as a negative control.

For HeLa (Figure 1A) and J774A.1 cells (Figure 1B), single time point assays showed a distinct trend, in which complex IV strains displayed higher levels of cytotoxicity than complex I isolates. For A549 cells the results were more dramatic (Figure 1C). Unlike other cell types previously examined [11,16,29], A549 cells are nearly resistant to cell death mediated by the RB50 T3SS (see RB50 vs. RB50Δ*bscN*; Figure 1C). Similarly, other complex I strains displayed little or no cytotoxicity against these cells. In striking contrast, incubation with complex IV isolates resulted in significant levels of cell death (p < 0.0001; Figure 1C). For A549 cells, strains D444 (ST15), D445 (ST17), D446 (ST3) and Bbr77 (ST18) were 10- to 15-fold more cytotoxic than RB50. Parallel assays measuring bacterial attachment to A549 cells did not detect significant differences between complex I and complex IV isolates, indicating that relative levels of adherence are not responsible for the observed differences in cytotoxicity (Additional file 1 Table S1).

**Figure 2:**
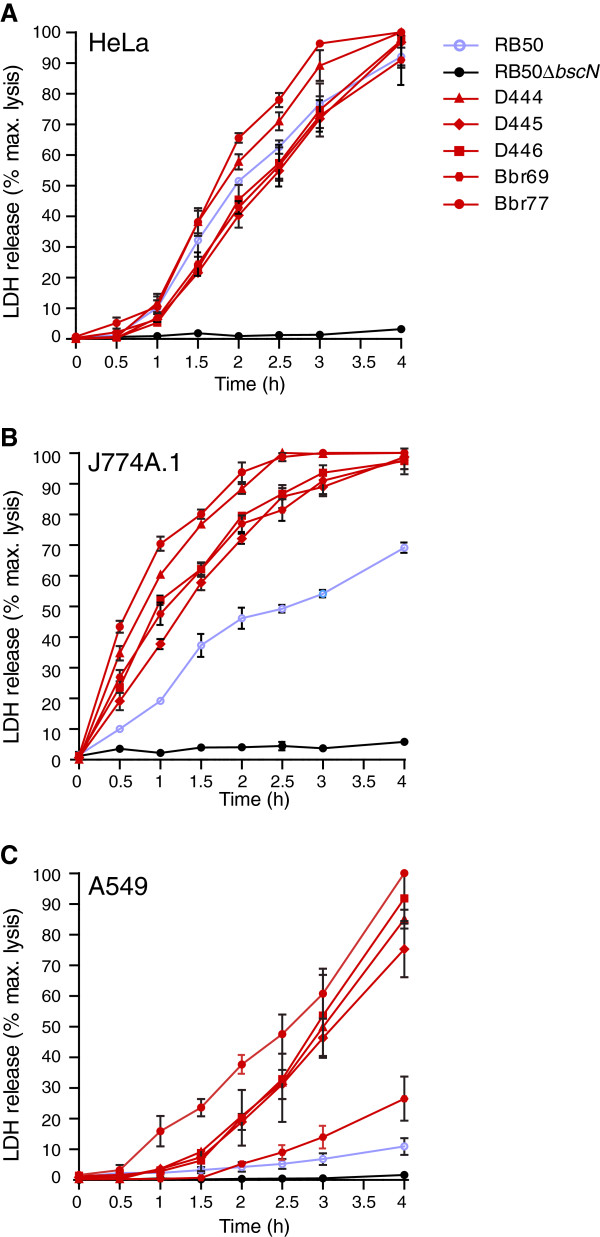
** Time course cytotoxicity assays. A**. HeLa, **B**. J774A.1, or **C**. A549 cells were infected with the indicated strains at a multiplicity of infection (MOI) of 50 in 12-well plates. Aliquots of culture supernatants were removed at the indicated times and lactate dehydrogenase (LDH) levels were measured as described in Materials and Methods. Complex I and complex IV strains are designated by blue or red lines, respectively. Due to repeated sampling of culture medium for LDH release assays, we consistently observe a slight increase in cytotoxicity measured in kinetic experiments *vs.* single time point assays as shown in Figure 1. The differences range from none to less than 20 %, depending on the cytotoxicity of the isolate. Error bars represent standard errors for measurements from at least three independent experiments.

Kinetic studies were performed next to increase the resolution of the analysis. We examined relative levels of cytotoxicity conferred by five complex IV strains towards HeLa, J774A.1 or A549 cells as a function of time, using RB50 as a complex I representative strain and RB50Δ*bscN* as a negative control. Following infection at an MOI of 50, cultures were sampled over a 4 h time period for measurements of LDH release. Using HeLa cells, which are exceptionally sensitive to T3SS-mediated killing, only minor differences were detected between strains (Figure 2A). Although two of the complex IV strains, Bbr77 and D444, displayed slightly elevated cytotoxicity at intermediate time points, all strains reached maximum lysis by the end of the 4 h time course. For J774 cells, differences between complex IV strains and RB50 were apparent throughout the experiment, with Bbr77 and D444 showing the highest levels of activity (Figure 2B). As expected, the most dramatic differences were seen with A549 cells (Figure 2C). Most complex IV strains displayed a marked hypercytotoxicity phenotype compared to RB50, with the exception of Bbr69 which had an intermediate phenotype. Interestingly, Bbr69 is a dog isolate whereas all of the other complex IV strains tested were cultured from human infections.

### Roles of the *bsc* T3SS and the BteA effector in hypercytotoxicity by complex IV *B. bronchiseptica* isolates

**Figure 3:**
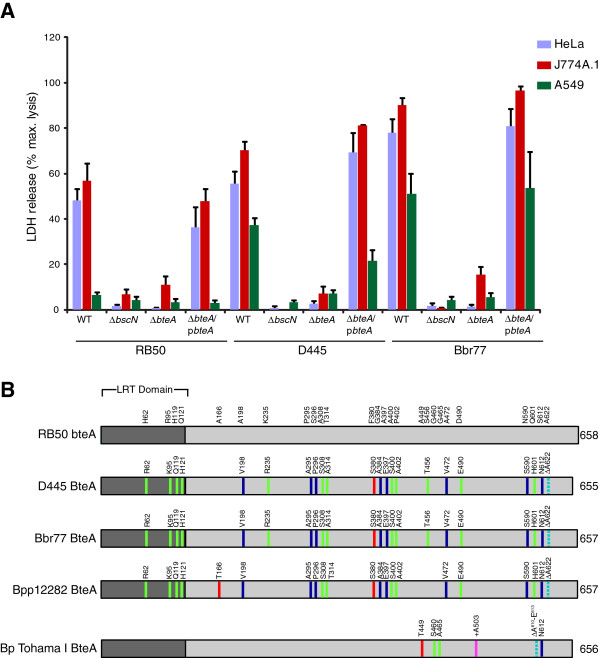
** Roles of the*bsc*T3SS and the BteA effector in cytotoxicity. A**. HeLa (blue bars), J774A.1 (red bars), or A549 cells (green bars) were infected with the indicated strains at a multiplicity of infection (MOI) of 50 in 24-well plates for 3 h. The *bteA* mutant strains were complemented in *trans* with the RB50 *bteA* allele carried on a medium copy vector (see Methods). Following infection, release of lactate dehydrogenase (LDH) into culture medium was measured as described in Methods. **B**. *bteA* homologues were compared using multialign [51] and amino acid differences are shown. Green lines indicate substitutions of highly conserved residues, blue shows weakly similar amino acids, red indicates no similarity, cyan dotted lines designate deletion of a residue and pink designates an amino acid insertion. Bp = *B. pertussis*, Bpp = *B. parapertussis*, LRT = lipid raft-targeting domain [12].

To examine the hypercytotoxicity phenotype in detail, two representative highly toxic complex IV strains of human origin, D445 (ST17) and Bbr77 (ST18), were chosen for further analysis. To measure the contribution of the *bsc* T3SS, nonpolar in-frame deletions were introduced into the *bscN* loci of D445 and Bbr77. As shown in Figure 3A*bscN* mutations eliminated *in vitro* cytotoxicity against all three cell types, demonstrating an essential role for type III secretion. We next examined the involvement of the BteA effector in hypercytotoxicity. Previous studies have shown that BteA is essential for T3SS-mediated cell death induced by RB50, and it is sufficient for cytotoxicity when expressed in mammalian cells [11]. For both complex IV strains, *bteA* deletion mutations had a similar effect as Δ*bscN* mutations and abrogated cytotoxicity (Figure 3A).

The BteA proteins expressed by Bbr77 and D445 are identical except for the absence of two amino acids at the extreme carboxyl end of D445 BteA (Figure 3B). In contrast, when compared to RB50 BteA, the complex IV effectors from Bbr77 and D445 differ at 22 or 24 positions, respectively (Figure 3B). Interestingly, BteA sequences from complex IV strains were more closely related to BteA in *B. parapertussis*_*hu*_Bpp12282 than to homologs in *B. bronchiseptica* RB50 or *B. pertussis* Tohama I. To determine whether BteA polymorphisms are responsible for differences in cytotoxicity phenotypes, *bteA* deletion derivatives of all three strains were complemented with the RB50 *bteA* allele on a medium copy vector (Figure 3A) [11]. In each case, complemented levels of cytotoxicity were similar to those of the wild type isolate. Most importantly, complemented Δ*bteA* derivatives of strains D445 and Bbr77 regained cytotoxicity against A549 cells, whereas RB50 Δ*bteA*/p*bteA* remained non-cytotoxic against this cell line. Taken together, these results demonstrate that the *bsc* T3SS and the BteA effector are essential for cytotoxicity by D445 and Bbr77. The hypercytotoxicity phenotypes of the complex IV isolates, however, are not due to polymorphisms in BteA. This is consistent with the conserved nature of this effector, both within and between *Bordetella* species [11]. Differential regulation of T3SS activity, the presence of novel secretion substrates, or alterations in accessory factors could account for phenotypic differences between strains (see Discussion).

### T3SS secretome analysis

We next compared polypeptide profiles of proteins secreted into culture supernatants by the isolates examined in Figure 3. Strains D445, Bbr77, and RB50 were grown to early mid-log phase in liquid medium under conditions permissive for type III secretion (Bvg + phase conditions, see Methods) [15]. To specifically identify T3SS substrates, Δ*bscN* derivatives were examined in parallel. Culture supernatants were TCA-precipitated, digested with trypsin, and separated by reverse-phase nano-liquid chromatography on a C18 column followed by tandem mass spectrometry (nLC-MSMS). Peptide profiles were queried against the RB50 protein database. Nearly identical sets of peptides were detected in supernatants from strains D445, Bbr77 and RB50, and these included peptides corresponding to T3SS substrates previously identified using RB50 (Table 2). Bsp22, which polymerizes to form an elongated needle tip complex [30], BopB and BopD, which form the plasma membrane translocation apparatus [14,29,31], BopN, a homolog of Yersinia YscN which functions as a secreted regulator [32], and the BteA effector were present in supernatants from wild type strains, but absent in supernatants of Δ*bscN* derivatives. In the course of this analysis we discovered a novel T3SS substrate encoded from a conserved hypothetical ORF (BB1639), herein named BtrA, in supernatant fractions from RB50, D445 and Bbr77 but not from their Δ*bscN* derivatives. Importantly, examination of complex IV secretion substrates failed to identify unique polypeptides that were not expressed by RB50 or did not match the RB50 protein database. The relative amounts of T3SS substrates released into culture supernatants, as assessed by SDS-PAGE and western blot analysis, also failed to correlate with relative levels of cytotoxicity (Additional file 2 Figure S1). Although these observations did not reveal obvious differences in the T3SS secretome that could account for the hypercytotoxic phenotypes of D445 and Bbr77, it is important to consider that the activity of the *bsc* T3SS and its substrate specificity are regulated at multiple levels, and results obtained using broth-grown cells provide only a crude approximation of T3SS activity during infection (see Discussion).

**Table 2:** nLC-MSMS secretome analysis

**Protein name**	**NCBI accession number**	**Sequence coverage (%)**					
		**RB50**	**RB50Δ*bscN***	**D445**	**D445Δ*bscN***	**Bbr77**	**Bbr77Δ*bscN***
Bsp22	gi|33568201	41	-	59	-	60	-
BopN	gi|33568200	24	-	29	-	24	-
BopB	gi|33568205	5	-	5	-	18	-
BopD	gi|33568204	50	-	51	-	54	-
BteA	gi|33568834	7	-	6	-	28	-
BtrA	gi|33568223	26	-	18	-	26	-

Summary of nLC-MSMS data indicated as peptide coverage for indicted T3SS substrate proteins in supernatant fractions from *B. bronchiseptica* strains grown to mid-log phase in Stainer-Scholte medium.

### Virulence of complex IV strains during respiratory infections

**Figure 4:**
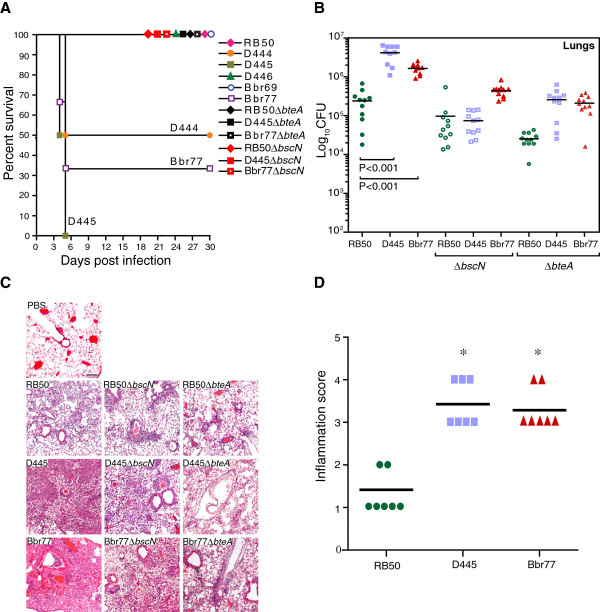
*** In vivo*characterization of selected complex IV*B. bronchiseptica*strains.A**. Survival of wild-type female C57BL/6NCr (B6) mice inoculated with different strains of *B. bronchiseptica*. Groups of four mice were intranasally inoculated with 5 x 10^5^ CFU of the indicated strains in 40 μl volumes as described in Methods. **B**. Female C57BL/6NCr (B6) mice were infected as above and sacrificed 3 days later. Lungs were removed, homogenized in sterile PBS, and aliquots were plated on selective media. The number of colony forming units (CFU) per lung is shown for each animal. **C**. Representative H&E-stained sections of lung tissue obtained on day 3 post infection with indicated strains (magnification, x5). **D**. Histopathological score of indicated strains based on criterion described in Methods. The * indicates *P* value of <0.0001 for RB50 vs. Bbr77 and RB50 vs. D445.

To determine if relative levels of cytotoxicity measured *in vitro* correlate with virulence *in vivo*, we used a murine respiratory intranasal challenge model [24]. Groups of 4–6 week old female specific-pathogen-free C57BL/6NCr mice were intranasally infected with 5 x 10^5^ CFU. At this dose, RB50 establishes nonlethal respiratory infections that generally peak around day 10 post-inoculation and are gradually cleared from the lower respiratory tract, while persisting in the nasal cavity [33].As shown in Figure 4A, complex IV strains segregated into two groups. The first caused lethal infections in some (D444, Bbr77) or all (D445) of the infected animals. The second group (D446, Bbr69) caused nonlethal infections similar to RB50.

In the experiment shown in Figure 4B, animals were intranasally inoculated with 5 x 10^5^ CFU of RB50 or the two most virulent complex IV isolates, D445 and Bbr77, and sacrificed three days later. Both complex IV isolates were present in lungs at levels that were 10 to 30-fold higher than RB50 (p < 0.001). Histopathological examination of lung tissue from mice infected with D445 or Bbr77 showed severe and widespread inflammation, affecting nearly the entire volume of the lung for D445 and up to 40% of the tissue for Bbr77 (Figure 4C & D). Extensive migration of lymphocytes, macrophages, and neutrophils resulted in severe consolidation of large areas of lung parenchyma. Alveolar and interstitial edema as well as extensive perivascular and peribronchiolar inflammation were also observed. In contrast, lungs from animals infected with RB50 displayed only mild inflammation that covered less than 25% of the total lung volume.

We also examined the relative roles of the *bsc* T3SS and the BteA effector in the *in vivo* virulence phenotypes of D445 and Bbr77. As shown in Figure 4A, deletions in *bscN* or *bteA* abrogated lethality following infection by either strain. Consistent with these observations, *ΔbscN* and Δ*bteA* mutants also showed significantly decreased numbers of bacteria in the lungs at day 3 post infection (Figure 4B) and a corresponding decrease in histopathology (Figure 4C). These results demonstrate that in comparison to the prototype complex I strain RB50, D445 and Bbr77 are more virulent in mice following respiratory infection, and hypervirulence is dependent on type III secretion and BteA.

### Comparative whole-genome analysis of complex I and complex IV *B. bronchiseptica* strains

**Figure 5:**
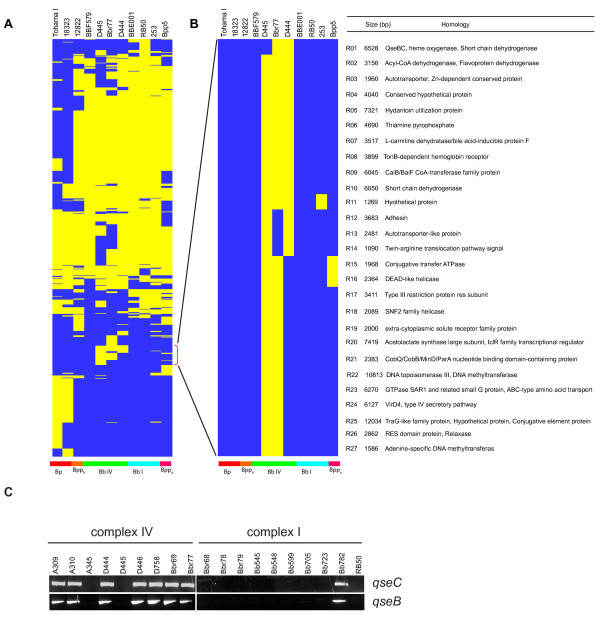
** Comparative genome analysis.A**. Cluster analysis of non-core genome sequences of 11 *Bordetella* strains. The results are displayed using TREEVIEW. Each row corresponds to a specific non-core region of the genome, and columns represent the analyzed strain. Yellow indicates presence while blue represents absence of particular genomic segments. Abbreviations: Bp = *B. pertussis*, Bpp_h_ = human *B. parapertussis*, Bb IV = complex IV *B. bronchiseptica*, Bb I = complex I *B. bronchisetpica*, Bpp_o_ = ovine *B. parapertussis*. **B**. Zoomed image of non-core region in panel A marked with a red bracket showing complex IV specific regions. On the right, blastn with default settings was used to query the nucleotide collection (nr/nt) from the National Center for Biotechnology Information and homology designations are indicated. **C**. Distribution of *qseBC* alleles among complex I and complex IV *B. bronchiseptica* isolates based on PCR-based amplification and sequencing.

To determine if hypervirulent complex IV *B. bronchiseptica* strains share common genomic regions that might be responsible for the phenotypes reported here, we obtained whole genomic sequences of D444 (MO149), Bbr77, and D445 using next-generation high throughput sequencing. We included in our analysis the genomic sequences of *B. bronchiseptica* strains BBE001 and 253 (complex I human isolates) [34,35], BBF559 (complex IV human isolate) [34], and RB50 [20]; *B. pertussis* strains Tohama I and 18323 [36]; and *B. parapertussis* strains 12822 and Bpp5 (human and ovine isolates, respectively) [37,38]. The *B. bronchiseptica* sequences were in various stages of assembly at the time of analysis (Table 3). Hierarchical clustering of virtual comparative genomic hybridization data supports previous MLST assignments of phylogenic relationships between *Bordetella* strains [10], as isolates from each complex are clustered together (Figure 5). Genome alignments reveal that these strains share approximately 2.5 Mb of "core" genome sequence.

**Table 3:** ***B. bronchiseptica*strains used for whole genome comparisons**

**Strain**	**Size (Mb)**	**ST (complex)**	**Contigs/Scaffold**
RB50	5.4	12 (I)	1
253	5.3	27 (I)	4
D444	5.1	15 (IV)	1
D445	5.2	17 (IV)	11
Bbr77	5.2	8 (IV)	16
BBE001	5.1	11 (I)	175
BBF579	4.9 (+IS481)	novel (IV)	319

We next carried out a comparative analysis of the non-core genome to identify potential loci shared only by complex IV strains. Despite sequences that are shared by more than one complex IV isolate, we did not identify complex IV genomic sequence(s) that *uniquely* differentiate complex IV from complex I strains. Strains D445, Bbr77 and D444 do, however, contain clusters of shared genes that are not present in other *Bordetella* genomes (Figure 5B, yellow boxes). Although these loci are missing in BBF579, the virulence properties of this isolate has not been reported, raising the possibility that one or more of these loci may contribute to hypervirulence by a subset of complex IV strains. Blastn analysis of overlap regions revealed a diverse set of genes involved mainly in signal transduction, metabolism, adhesin/autotransporter expression and type IV secretion of unknown substrates (Figure 5B).

One locus of potential interest, found in two out of four sequenced complex IV isolates (Bbr77 and D444) but none of the other *Bordetella* genomes, is predicted to encode homologs of the QseBC two-component regulatory system found in numerous bacterial pathogens [39]. In enterohemorrhagic *E. coli* (EHEC) and *Salmonella sp.*, QseBC has been shown to sense host stress hormones (epinephrine and norepinephrine) and regulate virulence gene expression [40-44]. The *qseBC* open reading frames from Bbr77 and D444 are identical, and their predicted products share 47% amino acid identity and 63% similarity with EHEC QseB, and 34% identity and 51% similarity with EHEC QseC, respectively. Using a PCR-based assay, we screened for the presence of *qseBC* in a larger collection of *B. bronchiseptica* isolates. As shown in Figure 5C, this locus is present in 7 out of 9 complex IV isolates, but only 1 out of 10 complex I isolates. Sequence analysis of PCR amplicons revealed high levels of nucleotide identity (> 97%) between *B. bronchisepticaqseBC* alleles. Although highly enriched in complex IV strains, *qseBC* is unlikely to represent a single, conserved pathway for hypervirulence since it is absent from strain D445. Nonetheless, the potential role of QseBC in *Bordetella*-host interactions warrants further study. In addition to examining gross genomic differences, we also analyzed polymorphisms in virulence loci. Nearly all of the virulence genes shared a high degree of homology (Additional file 3 Table S2). The *bsc* T3SS locus, the *btr* genes involved in T3SS regulation, as well as their upstream promoter regions had greater than 97% sequence conservation between RB50 and complex-IV strains. Additionally, our analysis confirms the absence of *ptx/ptl* loci and divergence in *tcfA* and *prn* genes in sequenced complex-IV isolates as previously described by Diavatopoulos et al. [10].

## Discussion

The existence of a distinct lineage of *B. bronchiseptica* strains associated with human infections was described several years ago [10]; however, little is known regarding the virulence properties of complex IV isolates or their epidemiological significance. Here we present evidence that complex IV isolates display significantly higher levels of cytotoxicity against a variety of cell lines *in vitro*. For a subset of complex IV strains that were isolated from humans with respiratory illness and represent distinct sequence types, we also demonstrate that hypercytotoxicity *in vitro* correlates with hypervirulence *in vivo*, and that both phenotypes are dependent on the *bsc* T3SS and the BteA effector.

To investigate the mechanistic basis for the quantitative differences in BteA-dependent cytotoxicity observed between complex I and complex IV strains, we took a genetic approach which is both simple and definitive. In the experiment in Figure 3A, we show that when the RB50 *bteA* allele is expressed in Δ*bteA* derivatives of RB50 *or* hypercytotoxic complex IV strains (D445 and Bbr77), the cytotoxicity profile of the parental strain is maintained. Thus, hypercytotoxicity is not due to differences in the specific activity of the *bteA* products. Additionally, the examination of culture supernatants also failed to detect differences in the T3SS secretome that could account for increased virulence. Although it is possible that one or more novel effectors that augment cytotoxicity are expressed by complex IV strains at levels that escape detection, it is also possible, and perhaps even likely, that differences in regulation are at play. We have previously shown that loci encoding *bteA* and *bsc* T3SS apparatus components and chaperones are regulated by the BvgAS phosphorelay through an alternative ECF-sigma factor, BtrS [11,23]. In addition to transcriptional control, the partner-switching proteins BtrU, BtrV and BtrW regulate the secretion machinery through a complex series of protein-protein interactions governed by serine phosphorylation and dephosphorylation [23,45]. Comparative expression analysis shows that differential expression of the BvgAS regulon correlates with human-adaptation by *B. pertussis* and *B. parapertussis*[18]. In a similar vein, it seems reasonable to suspect that T3SS regulatory systems may be adapting to the evolutionary pressures that are shaping *B. bronchiseptica* lineages.

Although both cytotoxicity and virulence are known, or likely, to be T3SS-dependent phenotypes in all strains examined, the correlation between lethality in mice and LDH release *in vitro* was not absolute. Strain D446 was highly cytotoxic to all cell lines examined (Figure 1), yet it was relatively avirulent following respiratory infection (Figure 4A). This is not unexpected given the fact that type III secretion is only one of many virulence determinants required for pathogenesis [7], and *B. bronchiseptica* isolates are known to have diverse phenotypic properties despite their high degree of genetic similarity. A recent study by Buboltz et al. [46] identified two complex I isolates belonging to ST32 which also appeared to have heightened virulence when compared to RB50. In particular, the LD_50_ of these strains was 40- to 60-fold lower than RB50 and based on transcriptome analyses, hypervirulence was associated with upregulated expression of T3SS genes. The authors also observed a T3SS-dependent increase in cytotoxicity towards cultured J774A.1 macrophage cells. It will be important to determine whether complex IV isolates do indeed share common virulence properties, or if the observations reported here represent heterogeneity distributed throughout *B. bronchiseptica* lineages.

Numerous studies have demonstrated the ability of the *bsc* T3SS to exert potent cytotoxicity against a remarkably broad range of mammalian cell types, regardless of their species or tissue of origin [11,12,14,15]. This was considered to be a defining feature of the *B. bronchiseptica* T3SS. A549 cells, derived from human alveolar epithelial cells, are the first cell line to our knowledge shown to be *resistant* to intoxication by RB50. The finding that complex IV isolates kill these cells with high efficiency provides particularly compelling evidence for their hypercytotoxicity*.*

To begin to address the comparative genomics of *B. bronchiseptica* lineages, we analyzed the genome sequences of 4 complex IV and 3 complex I strains. The observation that homologs of the *qseBC* locus are present in multiple complex IV strains was an intriguing discovery, as these genes encode a catecholamine-responsive virulence control system in *E. coli* and *Salmonella*[39-42]. Since the locus is missing in two complex IV strains (A345, D445), one of which is also hypervirulent (D445), *qseB* and *qseC* do not satisfy the criteria for either complex IV-specific or hypervirulence-associated genes. No loci were found to be uniquely present in all complex IV isolates, and we also failed to identify loci that are present in all members of the hypervirulent subset of complex IV strains and are predicted to encode factors involved in virulence. It is probable that there are multiple pathways to hypervirulence, and that polymorphisms between conserved virulence and regulatory genes play a role in this phenotype as well as the apparent predilection of complex IV isolates for human infectivity.

A particularly relevant question that remains to be addressed involves the burden of human disease currently caused by *B. bronchiseptica*. Diagnostic methods in common use that rely on PCR-based identification efficiently detect *B. pertussis* and *B. parapertussis*, but not *B. bronchiseptica*[47]. It is therefore possible that *B. bronchiseptica* respiratory infections are more common than previously appreciated, and it is intriguing to speculate that complex IV isolates may be responsible for undiagnosed respiratory infections in humans.

## Conclusions

This work provides an initial characterization of the virulence properties of human-associated *B. bronchiseptica.* In *in vitro* cytotoxicity assays using several mammalian cell lines, wild type complex IV isolates showed significantly increased cytotoxicity as compared to a panel of complex I strains. Some complex IV isolates were remarkably cytotoxic, resulting in LDH release levels that were 10- to 20-fold greater than the prototype complex I strain RB50. While infection of C57/BL6 mice with RB50 resulted in asymptomatic respiratory infection, a subset of complex IV strains displayed hypervirulence which was characterized by rapidly progressive pneumonia with massive peribronchiolitis, perivasculitis, and alveolitis. Although *in vitro* cytotoxicity and *in vivo* hypervirulence are both dependent upon T3SS activity and the BteA effector, the exact mechanistic basis for quantitative differences in cytotoxicity observed between complex I and complex IV *B. bronchiseptica* isolates is currently unresolved. A limited comparative genomic analysis did not reveal unique genetic determinants that could potentially explain the virulence phenotype associated with the complex IV isolates examined. Our observations of hypervirulence in tissue culture and animal models of infection suggests that further study of these potentially emerging human pathogens is warranted.

## Competing interests

The authors declare that there are no competing interests.

## Authors’ contributions

UA designed the study and was responsible for a majority of the experimental work, data interpretation, and writing of the manuscript. ML was responsible for the genome data analysis and revising the manuscript. ST, HL and JPark aided in genomic data analysis. PS and JW aided in MS data acquisition and analysis. JParkhill was responsible for genome data acquisition. ETH participated in data analysis and revision of the manuscript. JFM participated in study design, coordinated the study, and co-authored the manuscript. All authors reviewed and approved the final manuscript.

## Supplementary Material

Additional file 1 **Table S1.** Adherence of *B. bronchiseptica* isolates. HeLa or A549 cells were infected at a multiplicity of infection (MOI) of 200 in 12-well plates for 15 min. After infection, cells were washed with Hanks' balanced salts solution, fixed with methanol, stained with Giemsa stain and visualized by light microscopy. Adherence was quantified by counting the total number of bacteria per mammalian cell in at least three microscopic fields from two separate experiments. ++, 100-200 bacteria/cell; +, 1-100 bacteria/cell, -, no attachment, nd, not determined. Click here for file

Additional file 2 **Figure S1.** Secreted protein analysis of *B. bronchiseptica* isolates. Cultures were grown to late-log phase and pellet (0.125 OD_600_ equivalents) or supernatant (3.75 OD_600_ equivalents) fractions were separated by SDS-PAGE and stained with Coomassie brilliant blue. Molecular mass markers (kDa) are indicated on the left. Labels on the right show the identities of proteins determined by mass spectrometry. Click here for file

Additional file 3 **Table S2.** tBLASTn comparisons of known virulence genes. Values indicate % identity or % similarity at the amino acid level with respect to RB50. Click here for file
