# Supplementary material for: Phenotypic and Genomic Analysis of Hypervirulent Human-associated Bordetella bronchiseptica
Source: BMC Microbiol. 2012 Aug 6;12:167. doi: 10.1186/1471-2180-12-167 (PMC3462115; doi:10.1186/1471-2180-12-167)
Supplement: Additional file 1 — Table S1. Adherence of B. bronchiseptica isolates. HeLa or A549 cells were infected at a multiplicity of infection (MOI) of 200 in 12-well plates for 15 min. After infection, cells were washed with Hanks' balanced salts solution, fixed with methanol, stained with Giemsa stain and visualized by light microscopy. Adherence was quantified by counting the total number of bacteria per mammalian cell in at least three microscopic fields from two separate experiments. ++, 100-200 bacteria/cell; +, 1-100 bacteria/cell, -, no attachment, nd, not determined. [file 1471-2180-12-167-S1.docx]

| **Strain** | **HeLa** | **A549** |
| --- | --- | --- |
| Bbr69 | ++ | ++ |
| Bbr77 | ++ | ++ |
| D444 | ++ | ++ |
| D445 | ++ | ++ |
| D446 | ++ | ++ |
| RB50 | ++ | ++ |
| D758 | ++ | ++ |
| Bbr68 | ++ | ++ |
| Bbr78 | ++ | ++ |
| Bbr79 | ++ | ++ |
| Bb545 | ++ | ++ |
| Bb548 | ++ | + |
| Bb599 | ++ | ++ |
| Bb601 | ++ | ++ |
| Bb705 | ++ | ++ |
| Bb723 | ++ | ++ |
| Bb782 | ++ | ++ |
| RB54 | - | - |
| D445ΔbscN | nd | nd |
| D445ΔbteA | nd | nd |
| Bbr77ΔbscN | nd | nd |
| Bbr77ΔbteA | nd | nd |
| RB50∆bscN | nd | nd |
| RB50ΔbteA | nd | nd |
| RB50ΔbteA | nd | nd |

**Supplementary Table 1 Adherence of *B. bronchiseptica* isolates.**  HeLa or A549 cells were infected at a multiplicity of infection (MOI) of 200 in 12-well plates for 15min. After infection, cells were washed withHanks' balanced salts solution, fixed with methanol, stained with Giemsa stain and visualized by lightmicroscopy. Adherence was quantified by counting the total number of bacteria per mammalian cell in at least three microscopic fields from two separate experiments. ++, 100-200 bacteria/cell; +, 1-100 bacteria/cell, -, no attachment, nd, not determined.
