# Supplementary figures and images for: Phenotypic and Genomic Analysis of Hypervirulent Human-associated Bordetella bronchiseptica
Source: BMC Microbiol. 2012 Aug 6;12:167. doi: 10.1186/1471-2180-12-167 (PMC3462115; doi:10.1186/1471-2180-12-167)

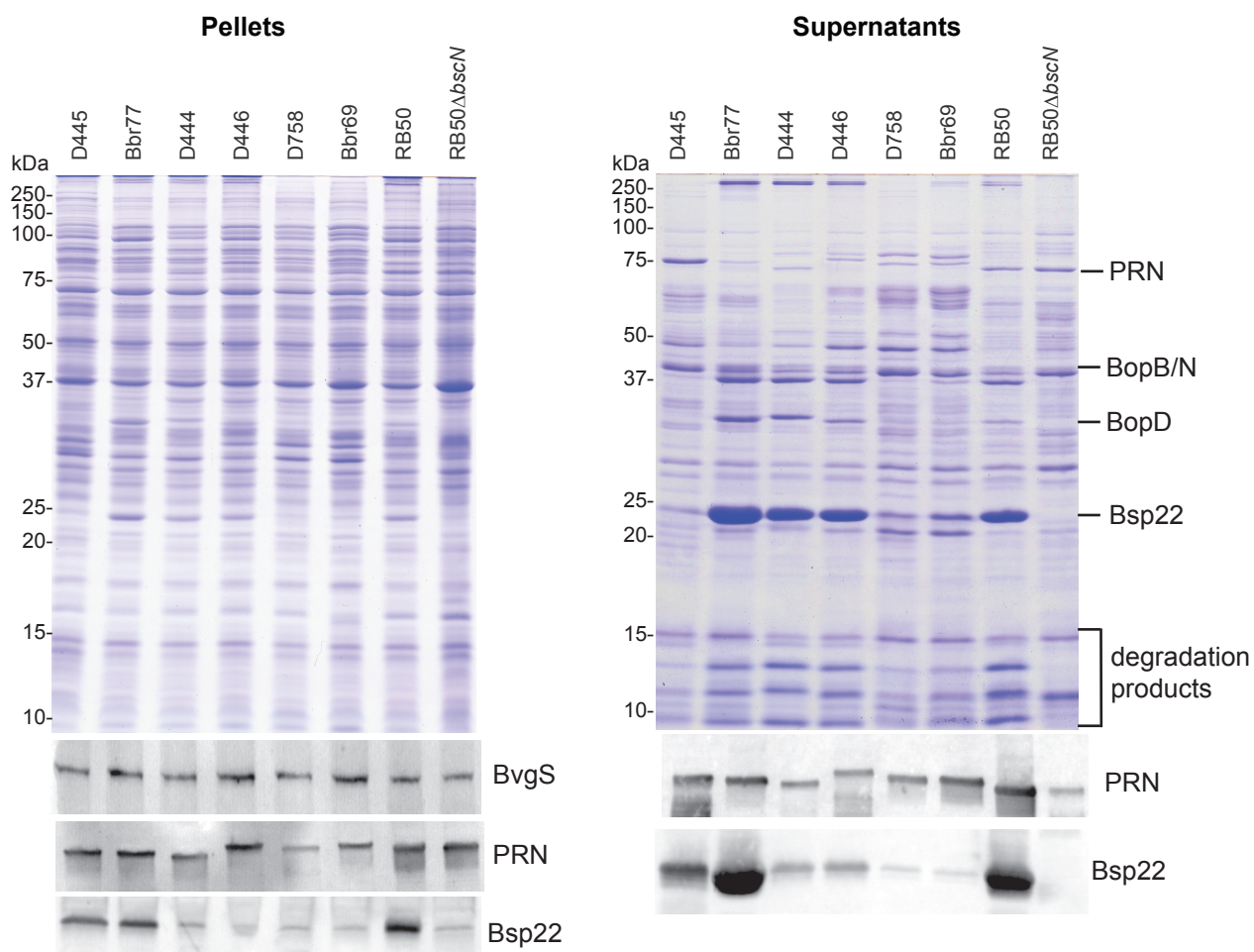

**Supplementary Figure 1 Secretome analysis of *B. bronchiseptica* isolates.**

Supplement: Additional file 2 — Figure S1. Secreted protein analysis of B. bronchiseptica isolates. Cultures were grown to late-log phase and pellet (0.125 OD600 equivalents) or supernatant (3.75 OD600 equivalents) fractions were separated by SDS-PAGE and stained with Coomassie brilliant blue. Molecular mass markers (kDa) are indicated on the left. Labels on the right show the identities of proteins determined by mass spectrometry. [file 1471-2180-12-167-S2.pdf]
