# Supplementary material for: Phenotypic and Genomic Analysis of Hypervirulent Human-associated Bordetella bronchiseptica
Source: BMC Microbiol. 2012 Aug 6;12:167. doi: 10.1186/1471-2180-12-167 (PMC3462115; doi:10.1186/1471-2180-12-167)
Supplement: Additional file 3 — Table S2. tBLASTn comparisons of known virulence genes. Values indicate % identity or % similarity at the amino acid level with respect to RB50. [file 1471-2180-12-167-S3.docx]

**Supplementary Table 2 tBLASTncomparisons of known virulence genes.**Values indicate%identity or %similarity at the amino acid levelwith respect to RB50.

| **Protein name (RB50)** | **D445**  (%identity/%similarity) | **Bbr77**  (%identity/%similarity) | **D444**  (%identity/%similarity) | **Bb253**  (%identity/%similarity) |
| --- | --- | --- | --- | --- |
| BB1609/BscF | 100/100 | 100/100 | 100/100 | 100/100 |
| BB1610/BscE | 98/100 | 98/100 | 98/100 | 100/100 |
| BB1611/BscD | 98/98 | 100/100 | 97/97 | 99/99 |
| BB1612/BcrD | 97/97 | 99/99 | 99/99 | 99/99 |
| BB1613 | 98/99 | 96/97 | 96/97 | 98/99 |
| BB1614 | 97/98 | 97/99 | 97/99 | 100/100 |
| BB1615 | 98/99 | 98/98 | 97/98 | 100/100 |
| BB1616/BopN | 99/99 | 99/99 | 99/99 | 99/99 |
| BB1617/Bsp22 | 96/98 | 96/99 | 96/99 | 100/100 |
| BB1618 | 94/95 | 96/97 | 96/97 | 98/99 |
| BB1619/BcrH1 | 96/96 | 95/96 | 96/96 | 99/99 |
| BB1620/BopD | 98/99 | 100/100 | 98/98 | 100/100 |
| BB1621/BopB | 97/99 | 97/98 | 97/99 | 99/100 |
| BB1622/BcrH2 | 100/100 | 100/100 | 100/100 | 100/100 |
| BB1623/Bcr4 | 99/99 | 99/99 | 99/99 | 100/100 |
| BB1624/BscI | 99/99 | 97/97 | 97/97 | 99/99 |
| BB1625/BscJ | 99/99 | 99/99 | 99/99 | 99/99 |
| BB1626/BscK | 98/99 | 98/98 | 98/99 | 98/99 |
| BB1627/BscL | 98/99 | 98/99 | 98/99 | 99/100 |
| BB1628/BscN | 99/99 | 99/99 | 99/99 | 99/99 |
| BB1629/BscO | 99/99 | 99/99 | 99/99 | 100/100 |
| BB1630/BscP | 100/100 | 96/96 | 97/98 | 100/100 |
| BB1631/bscQ | 97/98 | 98/98 | 98/98 | 98/99 |
| BB1632/BscR | 99/99 | 98/98 | 99/99 | 100/100 |
| BB1633/BscS | 100/100 | 100/100 | 100/100 | 100/100 |
| BB1634/BscT | 98/99 | 98/99 | 98/99 | 100/100 |
| BB1635/BscU | 99/99 | 100/100 | 99/99 | 99/99 |
| BB1636/BscW | 100/100 | 100/100 | 97/100 | 100/100 |
| BB1637/BscC | 98/99 | 98/99 | 99/99 | 99/99 |
| BB1638/BtrS | 100/100 | 100/100 | 100/100 | 100/100 |
| BB1642/BtrU | 98/99 | 99/99 | 99/99 | 100/100 |
| BB1645/BtrW | 100/100 | 100/100 | 100/100 | 100/100 |
| BB1646/BtrV | 98/100 | 98/100 | 98/100 | 100/100 |
| BB4227/BtcA | 99/99 | 99/99 | 98/99 | 100/100 |
| BB4228/BteA | 96/97 | 96/97 | 96/97 | 100/100 |
| BB2993/FhaB | 94/96 | 94/96 | 89/91 | 93/95 |
| BB2994/BvgA | 99/99 | 99/99 | 99/99 | 100/100 |
| BB2995/BvgS | 95/97 | 95/97 | 95/97 | 99/99 |
| BB2996/BvgR | 99/99 | 99/99 | 99/99 | 99/99 |
| BB0324/CyaA | 97/98 | 96/98 | 97/98 | low match |
| BB0325/CyaB | 99/99 | 99/99 | 99/99 | low match |
| BB0326/CyaD | 97/98 | 97/98 | 97/98 | low match |
| BB0327/CyaE | 98/100 | 95/97 | 98/98 | low match |
| BB0961 BrkA | 98/99 | 98/100 | 96/97 | 99/99 |
| BB0960/BrkB | 100/100 | 100/100 | 99/99 | 100/100 |
| BB1366/Pertactin | 93/96 | low match | 92/94 | 96/97 |
| BB0419/SphB1 | 98/99 | 98/00 | 98/99 | 100/100 |
| BB1864/Vag8 | 97/98 | 97/98 | 97/98 | 99/99 |
| BB3291/TcfA | low match | 66/78 | 75/89 | 99/99 |
| BB3674/Fim2 | 86/91 | 96/98 | 96/97 | 83/90 |
| BB1658/Fim3 | 96/98 | 96/99 | 96/99 | 98/99 |
| BB3426/FimX | 94/96 | 95/96 | 96/96 | 99/99 |
| BB2992/FimA | 99/99 | 99/99 | 99/99 | 100/100 |
| BB4890/PtxA | 95/96 | no match | no match | 97/98 |
| BB4891/PtxB | 88/92 | no match | no match | no match |
| BB4892/PtxD | 81/86 | no match | no match | 96/96 |
| BB4893/PtxC | 88/92 | no match | no match | 98/99 |
| BB4893/PtxE | 90/91 | no match | no match | 92/92 |
| BB4895/PtlA | 86/87 | no match | no match | 89/89 |
| BB4897/PtlC | 98/99 | no match | no match | 100/100 |
| BB4898/PtlD | 85/92 | no match | no match | 99/99 |
| BB4898A | 88/91 | no match | no match | 98/98 |
| BB4899/PtlE | 92/95 | no match | no match | 99/99 |
| BB4900/PtlF | 90/94 | no match | no match | 98/98 |
| BB4901/PtlG | 90/94 | no match | no match | 100/100 |
| BB4902/PtlH | 97/98 | low match | low match | 99/99 |
